# Functional Near-Infrared Spectroscopy Neurofeedback Enhances Human Spatial Memory

**DOI:** 10.3389/fnhum.2021.681193

**Published:** 2021-09-29

**Authors:** Xin Hou, Xiang Xiao, Yilong Gong, Zheng Li, Antao Chen, Chaozhe Zhu

**Affiliations:** ^1^State Key Laboratory of Cognitive Neuroscience and Learning, IDG/McGovern Institute for Brain Research, Beijing Normal University, Beijing, China; ^2^School of Education, Chongqing Normal University, Chongqing, China; ^3^State Key Laboratory of Cognitive Neuroscience and Learning, Center for Cognition and Neuroergonomics, Beijing Normal University at Zhuhai, Zhuhai, China; ^4^Center for Collaboration and Innovation in Brain and Learning Sciences, Beijing Normal University, Beijing, China; ^5^Key Laboratory of Cognition and Personality of the Ministry of Education, Faculty of Psychology, Southwest University, Chongqing, China

**Keywords:** neurofeedback, neuromodulation, fNIRS (functional near infrared spectroscopy), spatial memory, lateral parietal cortex

## Abstract

Spatial memory is an important cognitive function for human daily life and may present dysfunction or decline due to aging or clinical diseases. Functional near-infrared spectroscopy neurofeedback (fNIRS-NFB) is a promising neuromodulation technique with several special advantages that can be used to improve human cognitive functions by manipulating the neural activity of targeted brain regions or networks. In this pilot study, we intended to test the feasibility of fNIRS-NFB to enhance human spatial memory ability. The lateral parietal cortex, an accessible cortical region in the posterior medial hippocampal-cortical network that plays a crucial role in human spatial memory processing, was selected as the potential feedback target. A placebo-controlled fNIRS-NFB experiment was conducted to instruct individuals to regulate the neural activity in this region or an irrelevant control region. Experimental results showed that individuals learned to up-regulate the neural activity in the region of interest successfully. A significant increase in spatial memory performance was found after 8-session neurofeedback training in the experimental group but not in the control group. Furthermore, neurofeedback-induced neural activation increase correlated with spatial memory improvement. In summary, this study preliminarily demonstrated the feasibility of fNIRS-NFB to improve human spatial memory and has important implications for further applications.

## Introduction

Spatial memory is a form of memory responsible for encoding, storing, and retrieving information about spatial locations, configurations, or routes (De Renzi et al., 1977; Postma and De Haan, 1996; Kessels et al., 2001). This important cognitive function is of great relevance in our daily life, in which it enables us to remember the locations of objects or find our way about in the familiar environment. Spatial memory will decline with normal aging (Korman et al., 2019), and be impaired in several disease conditions, such as mild cognitive impairment (MCI), Alzheimer’s disease (AD), stroke, chronic stress, depression, and schizophrenia (Iachini et al., 2009). The decline or disruption in spatial memory will affect individuals’ normal life severely, make individuals dispirited, and reduce the life quality. Therefore, how to improve spatial memory ability and delay spatial memory loss in normal aging or disease state has critical meanings.

Neuromodulation techniques can be used to facilitate individuals’ cognition, behavior, and pathology by modifying the activity of specific neural targets. In contrast to exogenous brain modulation techniques, such as transcranial magnetic stimulation (TMS) and transcranial direct current stimulation (tDCS), neurofeedback is a relatively safe, side-effect-free, well-tolerated, and acceptable brain modulation technique (Luctkar-Flude and Groll, 2015). Neurofeedback is an endogenous form of neuromodulation technique involving a brain-compute interface (BCI) that maps the real-time neural signals to some form of feedback (usually visual or auditory stimuli), that allows individuals to arbitrarily manipulate the underlying neural activity (Robert and Evans, 2011; Ordikhani-Seyedlar et al., 2016; Fedotchev et al., 2017; Sitaram et al., 2017; Smetanin et al., 2018; Thibault et al., 2018; Al-Taleb et al., 2019). Neurofeedback is a promising noninvasive neuromodulation tool to change the neuroplasticity of target brain regions or networks, and then improve cognitive and behavioral functions involved in turn. The benefits of neurofeedback have been widely reported in clinical populations (deCharms et al., 2005; Subramanian et al., 2011, 2016; Lofthouse et al., 2012; Marx et al., 2015; Zotev et al., 2016; Young et al., 2017, 2018; Mehler et al., 2018; Carney, 2019; Kohl et al., 2019) and healthy individuals (Egner and Gruzelier, 2003; Gruzelier, 2009; Nan et al., 2012; Li et al., 2019; Zhao et al., 2019).

There are currently three main neuroimaging techniques to obtain the ongoing neural signal for further real-time feedback in neurofeedback: electroencephalography (EEG), functional magnetic resonance imaging (fMRI), and functional near-infrared spectroscopy (fNIRS). In contrast to MRI device, fNIRS device is much cheaper without extra running fees, relatively insensitive to head motion, has no special needs for performing place and fewer contraindications, and can be conducted in a more natural environment (Ferrari and Quaresima, 2012; Kober et al., 2014; Wriessnegger et al., 2017). Besides, fNIRS has a relatively higher and acceptable spatial resolution compared to EEG, which allows fNIRS-based neurofeedback (fNIRS-NFB) to achieve more precise self-regulation of a local cortical target (Kohl et al., 2020). As an emerging technique, fNIRS-NFB has been considered as an adequate alternative method to enhance human cognitive functions or behavioral performances, particularly for long-term and multi-session applications. This promising transcranial brain modulation technique has been successfully applied in many cognitive and behavioral areas, such as executive function (Hosseini et al., 2016), motor rehabilitation (Mihara et al., 2013), attention-deficit/hyperactivity disorder (Marx et al., 2015; Mayer et al., 2015), autism spectrum disorder (Liu et al., 2016), and social anxiety (Kimmig et al., 2019; for review see Kohl et al., 2020).

Spatial memory processing involves the posterior medial hippocampal-cortical network, mainly including the hippocampus, lateral parietal cortex (LPC), precuneus, posterior cingulate cortex, retrosplenial cortex, parahippocampal gyrus, and entorhinal cortex (Ranganath and Ritchey, 2012; Ritchey et al., 2015). The hippocampus is the core node of the posterior medial hippocampal-cortical network (Kahn et al., 2008), and plays an essential role in spatial and episodic memory processing (Bird and Burgess, 2008). The hippocampus has reciprocal connections with other regions in this network and receives the spatial representations input via the dorsal visual stream (Kahn et al., 2008). The LPC is part of the dorsal visual processing stream, extracts and integrates the spatial aspects from external visual inputs or internal mental representations (Sack, 2009; Seghier, 2013), then propagates the processed spatial context information to the hippocampus via the dorsal stream. The hippocampus will process the spatial information to more abstract allocentric spatial representations then combine them with non-spatial information (e.g., object, person) usually input via the ventral pathway to form the cognitive map (Tolman, 1948). Previous TMS studies showed that stimulation on LPC enhanced intrinsic functional connectivity within the related posterior medial hippocampal-cortical network (Wang et al., 2014; Hermiller et al., 2019) and increased task-induced activation of other connected regions in this network including the hippocampus (Kim et al., 2018). Spatial memory performance also increased after TMS stimulation over the LPC (Nilakantan et al., 2017). Therefore, in consideration of its critical role in spatial memory processing and accessibility for fNIRS, we believe that the LPC is a potential brain target for fNIRS-NFB to enhance human spatial memory.

Here, we intended to perform a pilot study to test whether fNIRS-NFB on the LPC could be used to improve human spatial memory performance. We expected that participants in the real experimental group would learn to successfully regulate the neural activity in the LPC, and that spatial memory performance would be increased after training.

## Materials and Methods

### Participants

This study was approved by Southwest University Brain Imaging Center Institutional Review Board. Fifty healthy college students (25 males, age range 18–25 years) were recruited from Southwest University (China) to participate in this study. All the participants were right-handed, had no mental or neurological disorders, did not take medications within the previous month. In the screening phase, any individuals with experience in mnemonic strategies or neurofeedback were excluded. All participants provided written informed consent and were compensated for their participation.

The participants were divided into two groups randomly: The experimental (total 30 participants, 15 males), and the active control group (total 20 participants, 10 males). All participants were blinded to the group allocation.

### Identification of Feedback Targets

**FIGURE 1:**
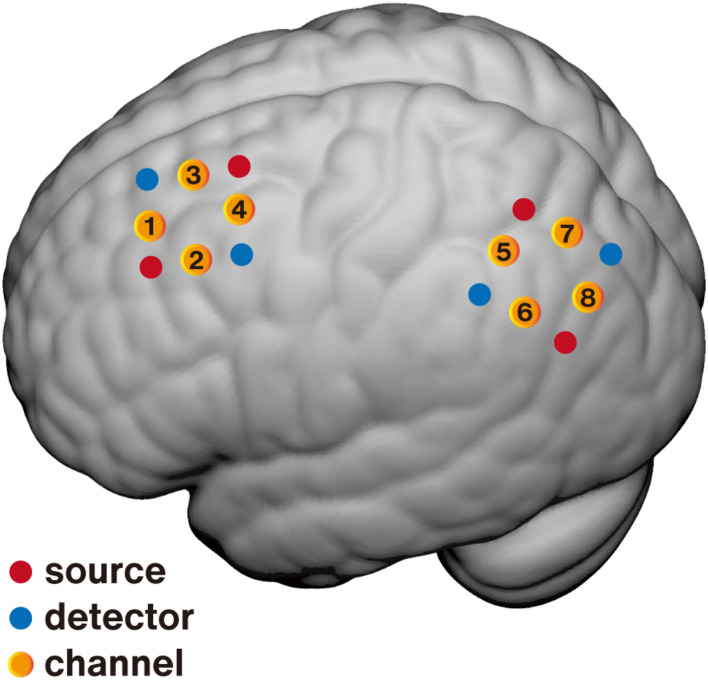
Schematic of fNIRS channel localization on the brain surface. Averaged positions for sources (red), detectors (blue), and channels (yellow) are overlaid on an MNI-152 canonical brain surface (MNI coordinates were generated using NFRI spatial registration toolbox developed by Singh et al., 2005). The premotor area (PMA, channel 4) was selected as the feedback target for the control group and the lateral parietal cortex (LPC, channel 8) for the experimental group.

Human spatial memory processing involves the posterior medial hippocampal-cortical network, especially the LPC and hippocampus. As described above, the LPC was selected as the potential neurofeedback target due to its important role in spatial cognitive processing and accessibility by fNIRS. While the LPC is a brain region with wide coverage and complex functions, it is important to identify a precise location within the LPC as the feedback target to achieve more specific and significant neural and behavioral effects for spatial memory. Previous studies have used resting-state functional connectivity to select the peak node within the LPC for its robust connection with the hippocampus as the TMS target (Wang et al., 2014; Kim et al., 2018), and the results have shown that the multi-day repetitive TMS (rTMS) stimulation on this region not only selectively enhanced the neural activation of related regions in the posterior medial hippocampal-cortical network including the LPC and hippocampus, but also significantly improved spatial memory performance (Nilakantan et al., 2017). Similar to this method, we also performed a resting-state functional connectivity analysis based on the open-access large sample fMRI database SLIM (Liu et al., 2017). A total of 112 participants with structural and resting-state MRI data (50 males, age range: 18–26 years, mean age = 20.7 years) were included in our analysis. The left middle hippocampus (3 mm radius sphere, center MNI: [–24, –18, –18]) was selected as the seed node, as it has robust connectivity with the cortical surface (Kahn et al., 2008). After preprocessing, a group-mean functional connectivity map was generated, multiple comparison corrected with FDR *p* < 0.005. Consistent with previous studies (Kahn et al., 2008), the left LPC (mainly including the angular gyrus, peak voxel MNI: [–45, –69, 33]) exhibited robust positive connectivity with the hippocampus. Then, the accessible superficial MNI coordinates within the hippocampal functional connectivity mask were projected to the scalp, and a transcranial brain atlas-based magnetic navigation system (Xiao et al., 2018) was used to assist in placing the fNIRS channels to cover the regions of interest (Figure 1).

Control conditions are essential for neurofeedback studies to distinguish whether neuropsychological changes were due to regulation of the target region or placebo effects. A control condition wherein participants receive contingent feedback from the irrelevant area(s) is recommended for determining the specificity of neurofeedback (Sulzer et al., 2013; Sorger et al., 2018; Thibault et al., 2018). The movement-related cortical region from a low-level system irrelative with the spatial memory-related network is more suitable for the control condition in this study. Besides, to minimize the contralateral confusion, the left hemisphere was selected. Finally, the left PMA (mean MNI [–38, –13, 60]) was selected as the feedback target for the active control group (Figure 1).

### Neurofeedback Protocols

**FIGURE 2:**
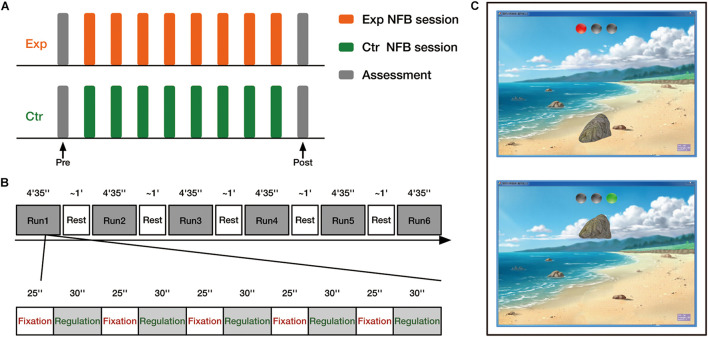
Experimental design overview. **(A)** Experimental procedure. Both experimental group (Exp) and control group (Ctr) received 8 neurofeedback (NFB) sessions. Before and after training, all participants completed the behavioral assessments. **(B)** Timeline of one neurofeedback session. **(C)** Neurofeedback visual interface. Two sample images of the interface depict the fixation (upper) and regulation (lower) blocks.

The experiment was divided into three stages: pre-assessment, 8 fNIRS-NFB sessions, and post-assessment (Figure 2A). The day before the first feedback session, the pre-assessment was performed to assess the baseline behavioral performance. Then, each participant received 8 neurofeedback sessions within 9 days. Each session had about 30 min of effective feedback time. There was 1 day without training between the first and last feedback sessions to avoid absence. Post-assessment was performed on the day after the final feedback session.

Changes in oxyhemoglobin concentration (HbO) were measured by the NIRS system (FOIRE-3000, Shimadzu Corporation, Kyoto, Japan) with two 2 × 2 probe sets designed to cover the feedback brain regions of interest. The probe arrays allowed for 8 different measurement channels, with 3.0 cm of source-detector separation. Neurofeedback was performed on our in-house fNIRS neurofeedback platform, which has previously been used to improve individuals’ cognitive flexibility (Li et al., 2019). Each neurofeedback session comprised 6 runs (4 min 35 s per run) separated by 5 short breaks. Each run consisted of five fixation blocks (25 s/ block) and regulation blocks (30 s/ block), starting with a fixation block (Figure 2B).

The neurofeedback interface (Figure 2C) presented to participants was a view of a sandy beach with a large stone that could be moved in a vertical direction. There were three gray indicator lights in the upper part of the screen. After a transient prompt tone, the left light turned red, indicating the start of the fixation block. During fixation blocks, the stone was kept static on the sand, and participants were asked to passively look at the stone and relax without thinking of anything. Subsequently, the right light turned green along with another transient beep, indicating the start of the regulation block. In this phase, participants were instructed to raise the stone by up-regulating the neural activity of their target brain region. Given that the explicit strategy instruction is not necessary for successful regulation (Sepulveda et al., 2016; Thibault et al., 2018), no explicit strategies were provided. Participants were asked to raise the stone as high as they could by using any mental strategy they found helpful. Large head motions and intentional breath control were not allowed because of the large influence on the fNIRS recordings.

**FIGURE 3:**
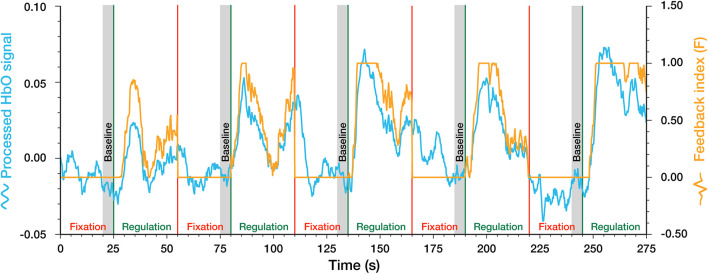
Example of the processed HbO signal and feedback index in a complete regulation run.

The ongoing raw signal was received by the neurofeedback platform from the fNIRS recording device at a high sampling rate (1/0.012 Hz). The raw signals were firstly down-sampled to a lower rate (1/0.28 Hz) and were then smoothed using a 1-s wide sliding window moving average filter. To calculate the real-time feedback index, a common method—the relative amplitude change value (Marx et al., 2015; Barth et al., 2016; Hudak et al., 2017, 2018)—was used to calculate the feedback index. The mean value of HbO in the last 5 s during fixation block before the following regulation block was firstly calculated as the baseline (Marx et al., 2015; Hudak et al., 2017). During regulation blocks, the preceding baseline was subtracted from the ongoing signal at each time point and was divided by a study-specific “difficulty coefficient” *M* to get the relative brain activity change value (*f*). The “difficulty coefficient” serves to calibrate the feedback visualization according to the variation range of the feedback signals to promote learning success (Li et al., 2019). Here, the “difficulty coefficient” *M* was set at 0.05 based on our experience in the pre-experiment. Then this relative brain activity change value (*f*) was transformed into the final feedback index (*F*) using the following formula which essentially limits the range from 0 to 1 (see following equations and Figure 3). The continuous neurofeedback index (*F*) was presented to participants in real-time via a movable stone on a 0–1 scale from the ground to the top of the screen (see Figure 2C).


$$\begin{array}{r} f=\frac{HbO_{reg}-Mean(HbO_{base})}{M}\\ F=\{\begin{array}{rr} 0, & f\leq 0;\\ f, & 0<f<1;\\ 1, & f\geq 1; \end{array} \end{array}$$

### Behavioral Assessment

To measure the behavioral effects induced by fNIRS-NFB, all participants performed an associative object-location memory task in the pre- and post-assessment sessions, which is a common task to measure human spatial memory ability (Nilakantan et al., 2017; Tambini et al., 2017, 2018). There were two sets (A and B) of spatial memory tests. To control for order effects, half of the participants of the experimental and control groups performed the set A in pre-assessment, and then the set B in post-assessment. The other half of each group used the tests in the opposite order. Each object stimuli set consisted of 25 unique and common object line drawings. The high-quality colorful object images were randomly selected from the Multilingual Picture (MultiPic) databank^1^ that is a set of publicly available 750 drawings from common concrete concepts (Duñabeitia et al., 2018). The stimuli presentation and response recording were implemented through the Psychtoolbox-3^2^ on Matlab (R2012a).

**FIGURE 4:**
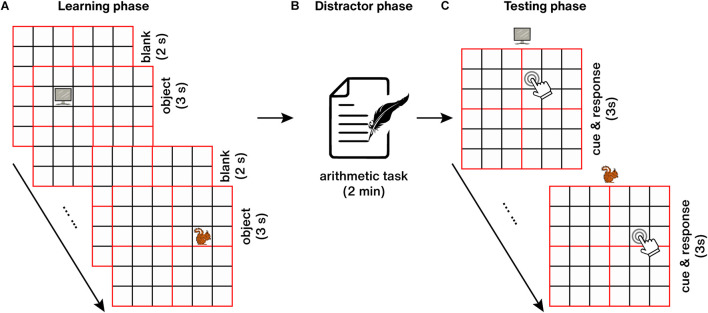
Object-location spatial memory task. **(A)** Learning phase. **(B)** Distractor phase. **(C)** Testing phase.

The entire spatial memory testing process consisted of three parts: learning phase, distractor phase, and testing phase (Figure 4). During the learning phase (Figure 4A), 25 objects were presented at randomized locations (the center of each square) on an empty 6 × 6 grid. Every object was presented for 3 s, and the inter-trial interval (ITI) was 2 s. Participants were instructed to study the object-location associations. Immediately after the learning phase, a 2-min arithmetic distractor task was followed to prevent further rehearsal or elaboration of the learned object-location associations (Figure 4B). After that, the testing phase started (Figure 4C), in which each object was presented above an empty grid, and participants were asked to recall the paired location learned previously as accurately as possible, then move and click on the mouse to its final location (any position within the corresponding square) within 3 s.

Besides the spatial memory processing, the LPC, as part of a more global neural network, is involved in several general cognitive functions, such as working memory (Tumati et al., 2019). In order to test whether the spatial memory enhancement was induced by neural feedback training on the specific target rather than other general cognitive function improvements, we also performed a classical sequential letter n-back test (Lieberman and Rosenthal, 2001) to test the working memory ability (memory load *n* = 3 in this study).

### Data Analysis

#### Neural Data Analysis

The recorded raw fNIRS neurofeedback data were processed and analyzed offline using an open-source software NIRS-KIT (Hou et al., 2021). All the raw fNIRS signals were visually inspected throughout the experiment to control the signal quality. The subjects with severe head motion in more than 2 bad sessions (≥ 3 bad runs in each session) or 3 bad sessions (≥ 2 bad runs in each session) were removed from further analysis.

After quality control, the survived fNIRS data were preprocessed to minimize the influence of noise and artifacts. First, the linear baseline drifts due to long-term physiological shifts or instrumental instability were removed by using a first-order detrending. Since measurement optodes are typically affixed to the surface of the head using a cap, fNIRS is more tolerant of head motion than fMRI. However, NIRS is not impervious to head-motion-induced noise. When severe head movements occurred that may be caused by sudden or unconscious movements, the NIRS optodes (source or detector) may shift relative to the head and alter the coupling between the optodes and scalp (Cui et al., 2010). This will result in an artifact that changes abruptly with the motion, which is more likely to degrade signal quality (Brigadoi et al., 2014). Therefore, despite the relative insensitivity of fNIRS to head motion, it is still necessary to apply preprocessing strategies that eliminate artifacts resulting from excessive head movement (Fishburn et al., 2019). Here, a temporal derivative distribution repair (TDDR) algorithm was used for head motion correction (Fishburn et al., 2019). Then, a Butterworth band-pass filter (third order, 0.0078–0.01 Hz) was applied to remove the irrelevant low-frequency and high-frequency components.

To estimate the effect of neurofeedback on cortical activation, a general linear model (GLM) was applied on the individual level (Fujimoto et al., 2017; Li et al., 2019). In the GLM model, the task condition for regulation periods was convolved with a standard canonical hemodynamic response function (HRF) to form the corresponding regressor, and the rest periods were included as the implicit baseline. Then, individual neurofeedback-induced (NFB-induced) activation (β: beta value) for each measurement channel was evaluated.

The individual activation values within every session were firstly averaged. To explore whether subjects learned to regulate the neural activity in the region of interest successfully, mixed ANOVAs, with between-participants factor (group) and within-participants factor (time), were performed. Then, paired *t*-test was performed to compare the regulation-induced activation values of each group between the following sessions and the first session for *post hoc* analysis.

#### Behavioral Data Analysis

To better characterize the spatial memory ability, we calculated the recollection precision, which is more sensitive to the recollected quality (Harlow and Yonelinas, 2016; Nilakantan et al., 2018) and subtle performance change. Similar to previous studies, the error distance (*Ed*_*i*_) between each object’s response location during the memory test and its target location during the learning phase was introduced to compute the spatial memory precision (Nilakantan et al., 2017, 2018; Tambini et al., 2017). Then, the value was normalized to the scale of [0∼1] to represent the spatial memory recollection precision:$Precision=\frac{MaxEd_{i}-Ed_{i}}{MaxEd_{i}}$ (where *MaxEd*_*i*_ denotes the maximal possible error distance for each object).

To test whether fNIRS-NFB improved individuals’ spatial memory performance, paired *t*-test (pre vs. post) were used for each group. Then, a two-sample *t*-test was performed to test whether the group difference of the behavioral effects was significant. Similar analyses were also performed on working memory accuracy.

Besides, to detect the relationship between the neurofeedback-induced neural effects and behavioral effects, Pearson correlation analysis was performed. Pearson correlation analysis was also conducted to test whether working memory change contributes to the spatial memory improvement induced by fNIRS-NFB.

Statistical analysis for neural and behavioral data was performed using MATLAB (R2012a) and SPSS (IBM Crop., version 25). The significance threshold was set to α = 0.05 (two-sided).

## Results

As stated above, one participant in the experimental group and two participants in the control group were excluded because of severe head motion artifacts. Therefore, the following results were based on the final participant set consisting of 29 participants in the experimental group and 18 participants in the control group. We found no significant age difference between the two groups [*t*_(45)_ = 0.98, *p* = 0.333, Cohen’s *d* = 0.29, see Table 1]. No significant group difference was found in either spatial memory precision [*t*_(45)_ = 0.49, *p* = 0.625, Cohen’s *d* = 0.15] or working memory accuracy in pre-assessment [*t*_(45)_ = 0.68, *p* = 0.54, Cohen’s *d* = 0.20] in the pre-assessment.

**TABLE 1:** Demographic information and spatial memory performance.

Variables	Experimental (*n* = 29)		Control (*n* = 18)		Group difference *t*-value (pre)	Group difference *t*-value (post—pre)
	Pre	Post	Pre	Post		
Gender (male)	14		9			
Age (year)	21.1_(0.78)_		21.5_(0.37)_		0.98	
Spatial memory	0.79_(0.06)_	0.86_(0.04)_***	0.80_(0.05)_	0.79_(0.05)_	0.49	3.02**
Working memory	0.77_(0.09)_	0.83_(0.08)_***	0.74_(0.08)_	0.83_(0.08)_**	0.68	0.82

Entries are means (SEM). **p < 0.01; ***p < 0.001.

### Neural Results

**FIGURE 5:**
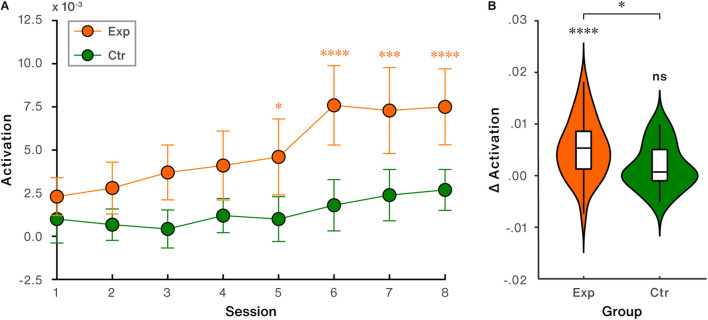
NFB-induced activation performance in the target region (channel 8: lateral parietal cortex). **(A)** Trends of NFB-induced activation across all sessions. Error bars indicate standard error of the mean (SEM). **(B)** NFB-induced activation changes (last session vs. first session). ^∗^*p* < 0.05; ^∗∗∗^*p* < 0.001; ^∗∗∗∗^*p* < 0.0001; ns, non-significant.

The NFB-induced activation in the region of interest for each session was calculated and presented in Figure 5A. To evaluate the training success on the neural level in the target channel, a mixed two-way ANOVA with within-subject factor (time: session1/session2/…/session8) and between-subject factor (group: experimental group vs. control group) was conducted. The results showed significant main effects of time [*F*_(7, 315)_ = 10.74, *p* < 0.001, η^2^ = .19] and group [*F*_(1, 45)_ = 7.83, *p* = 0.008, η^2^ = 0.15]. The interaction between time and group was significant [*F*_(7, 315)_ = 2.78, *p* = 0.008, η^2^ = 0.06]. *Post-hoc* comparisons demonstrated that activation in the target channel significantly increased by session 5 and the following sessions vs. the first session in the experimental group (session 5 > session 1, *p* = 0.040; session 6 > session 1, *p* < 0.0001; session 7 > session 1, *p* < 0.0001; session 8 > session 1, *p* < 0.0001). But not significant increases were found in the control group (all *p*s > 0.109). Direct inter-group comparison further revealed that the experimental group exhibited significantly higher activity compared to the control group during session 3 (*p* = 0.018) and sessions 5∼8 (session 5: *p* = 0.037; session 6: *p* = 0.002; session 7: *p* = 0.013; session 8: *p* = 0.007).

Directly comparing the NFB-induced activation change (last session vs. first session) in LPC revealed that significant increase was found in the experimental [*t*_(28)_ = 4.83, *p* < 0.0001, Cohen’s *d* = 0.90], but not in the control group [*t*_(17)_ = 1.65, *p* = 0.117, Cohen’s *d* = 0.39], shown in Figure 5B. Between-group comparison showed that the difference of regulation-induced activation change in LPC was significant [*t*_(45)_ = 2.21, *p* = 0.033, Cohen’s *d* = 0.66, Figure 5B].

### Behavioral Results

**FIGURE 6:**
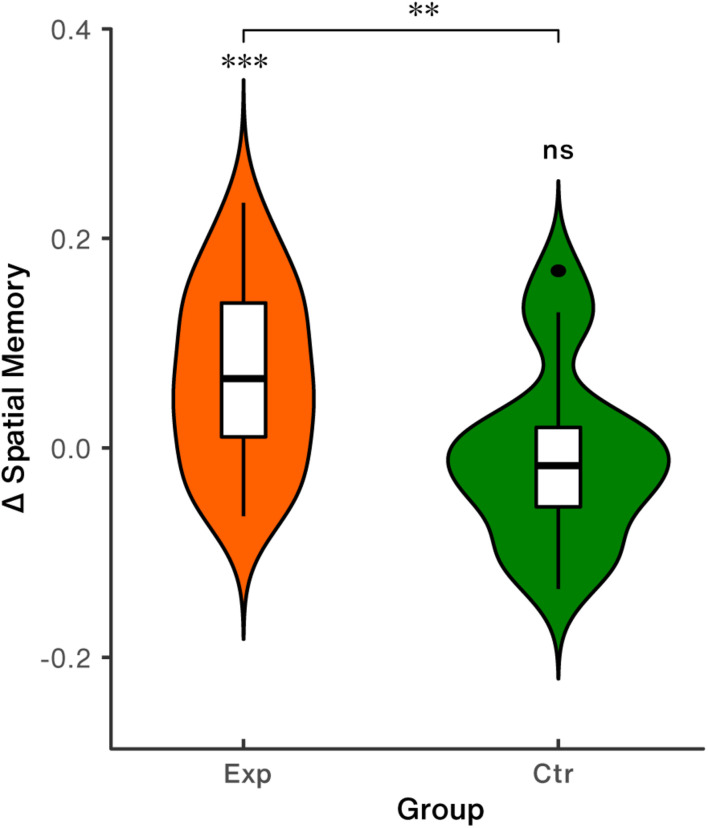
Spatial memory performance. Exp: the experimental group; Ctr: the control group; ^∗∗^*p* < 0.01; ^∗∗∗^*p* < 0.001; ns, non-significant.

Behavioral effect analyses showed that spatial memory precision significantly increased relative to baseline in the experimental group [*t*_(28)_ = 4.24, *p* < 0.001, Cohen’s d = 0.79], while the change was not found to be significant in the control group [*t*_(17)_ = 0.47, *p* = 0.642, Cohen’s d = 0.11], as shown in Table 1 and Figure 6. Group difference of NFB-induced behavioral changes was significant [*t*_(45)_ = 3.02, *p* = 0.004, Cohen’s *d* = 0.90, Figure 6].

Control behavioral analysis showed that significant increases of working memory accuracy were obtained not only in the experimental group [*t*_(28)_ = 3.91, *p* < 0.001, Cohen’s *d* = 0.73], but also in the control group [*t*_(17)_ = 3.391, *p* = 0.004, Cohen’s *d* = 0.80]. And group difference of working memory gain was not significant [*t*_(45)_ = 0.82, *p* = 0.416, Cohen’s *d* = 0.25, see Table 1]. These results indicate that there is no selectivity in increasing working memory performance after training.

### Relationship Between Neural and Behavioral Effects

**FIGURE 7:**
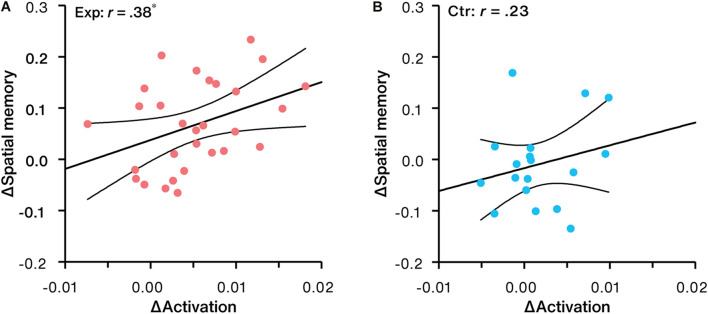
Correlation between NFB-induced activation change in LPC and spatial memory change for the **(A)** experimental group (Exp) and **(B)** control group (Ctr). ^∗^*p* < 0.05.

Correlation analysis revealed that NFB-induced activation changes in LPC were significantly and positively correlated with increases in spatial memory performance in the experimental group (*r* = 0.38, *p* = 0.040, 95% CI: [0.02, 0.66], Figure 7A). No significant trend (*r* = 0.23, *p* = 0.352, 95% CI: [–0.26, 0.63], Figure 7B) was found in the control group.

No significant correlation trends between spatial memory improvements and working memory changes were obtained in both groups (*r* = 0.25, *p* = 0.197, 95% CI: [–0.13, 0.56] for the experimental group; *r* = 0.27, *p* = 0.270, 95% CI: [–0.22, 0.66] for the control group).

## Discussion

In this pilot study, fNIRS-NFB was preliminarily used to allow individuals to manipulate the neural activity of LPC with the aim of improving human spatial memory performance. The results showed that participants in the experimental group but not in the control group learned to regulate the neural activation in this cortical target successfully accompanied by significantly increased spatial memory performance. Furthermore, increase of NFB-induced activation in LPC could predict the improvement in spatial memory performance. This pilot trial preliminarily verified the feasibility of fNIRS-NFB to improve human spatial memory by modulating the related cortical region.

Here, fNIRS-NFB was used to assist individuals to voluntarily modulate the neural activity of LPC, a cortical region for spatial memory processing, with the aim to improve human spatial memory performance. In the experimental group, real-time signals of interest from the target region were fed back, while subjects in the control group received the real-time feedback neural signals for an irrelevant cortical region. The neural effects analysis results showed that LPC activity significantly increased over the last four training sessions relative to the first session in the experimental group, but no significant changes were observed in the control group. The successful regulation effect on the neural level was further confirmed by between-group differences analysis. These target-specific regulation effects on the neural level demonstrated that only successful regulation on specific regions of interest can produce the desired neural changes. On the behavioral level, spatial memory performance increased significantly in the experimental group but not in the control group. Moreover, the selective behavioral increase was only observed in spatial memory performances rather than in working memory changes. This suggests that significant increases in working memory in both groups might be caused by certain unspecific factors, such as practice effect rather than specific neurofeedback training. Importantly, correlation analyses revealed that the increases in LPC activation rather than changes in working memory performance can predict the improvements of spatial memory performance, further confirming a potential functional relevance between successful LPC regulation via fNIRS-NFB and the following behavioral effects on spatial memory improvement. It is important to be noted that though working memory as a general cognitive function has been measured to demonstrate the specificity on behavioral effects, other general cognitive functions involved by LPC and related brain networks, such as attention (Cabeza et al., 2012; Tumati et al., 2019), should also be tested in the future to further verify the specificity of the fNIRS-NFB.

In the current study, increased NFB-induced activation in LPC during regulation was significantly associated with spatial memory improvement, which suggests that improved function in this region makes contributions to better spatial memory performance. The LPC is involved in a wide range of tasks in spatial cognition, which reflects its ability to extract and integrate the spatial aspects of our environment, including the spatial analysis of external sensory information or internal mental representations (Sack, 2009; Seghier, 2013; Tumati et al., 2019). Previous studies have demonstrated that it enables the rich and vivid subjective experience during episodic memory retrieval, which benefits successful and precise recollection (Ciaramelli et al., 2008; Vilberg and Rugg, 2008). Furthermore, exogenous neuromodulation studies showed that high-frequency rTMS applied over the LPC enhanced object-location recollection (Nilakantan et al., 2017; Kim et al., 2018). Combined with the previous evidence, the current results may suggest that the improved functions induced by neurofeedback in the LPC would support better, vivid, and precise spatial-related information processing, and result in the better spatial memory performance.

Besides the direct local effects, the potential remote neural effects in other related brain regions caused by fNIRS-NFB might also contribute to the final behavioral improvement. The neurofeedback cortical target, LPC, has reciprocal connections with other regions in the posterior medial network and projects to the medial temporal lobe (MTL). With robust structural and functional connections to LPC (Kahn et al., 2008), the hippocampus receives input from LPC likely mediated by the lateral parietal projections to the retrosplenial and parahippocampal cortex (Mesulam et al., 1977; Mufson and Pandya, 1984; Cavada and Goldman-Rakic, 1989). The hippocampus is the core component of the distributed hippocampal-cortical network (Battaglia et al., 2011; Ranganath and Ritchey, 2012), which plays a crucial role in spatial memory processing (Bird and Burgess, 2008). For spatial memory processing, the hippocampus receives the spatial context information input (from LPC) via the dorsal pathway and the non-spatial information (e.g., object, person) usually input via the ventral pathway, then combines the item-context association to form the cognitive map, and will be reactivated to extract the item-context association during retrieval (Tolman, 1948). Previous studies showed that TMS stimulation over LPC affects the remote and related brain regions, especially the hippocampus (Wang et al., 2014; Kim et al., 2018; Tambini et al., 2018; Freedberg et al., 2019). Based on the above findings, we speculate that fNIRS-NFB of LPC might enhance spatial memory performance via the potential neural influences both on LPC and on the related network regions, especially the hippocampus.

The polity fNIRS-NFB study has potential practical and social values. Compared with other brain modulation techniques, fNIRS-NFB has several special advantages including perfect safety, low cost, simplicity, portability, accessibility, and wide applicability, which makes it a promising brain modulation tool. Given these special advantages and the positive results in the current study, fNIRS-NFB has great potential to be widely applied in future clinical and non-clinical situations for patients and elderly people who are suffering spatial memory loss or decline.

However, the results of the current study should be interpreted in line with its limitations. First, this pilot study was performed based on a limited sample size and a healthy young adult population sample, which somewhat limits the generalization of this study. Future studies are needed to test its feasibility in larger sample sizes and different populations to further verify its effectiveness and feasibility, especially in the clinical patients and elderly population. Second, it is important to know whether the learning effects of self-regulation can be maintained over longer periods beyond the initial training period, especially for clinical applications. Although several previous studies have reported that the neural and behavioral effects induced by neurofeedback could persist for weeks or even months after intervention (Marx et al., 2015; Robineau et al., 2017; Rance et al., 2018; Van Doren et al., 2019), the special time course of the lasting effects induced by fNIRS-NFB has not been characterized in the current study. We recommend that future studies should perform regular follow-up measurements for weeks or months after the intervention, which will further verify the feasibility of the study. Besides, the underlying neural mechanism caused by fNIRS-NFB which contributes to final spatial memory improvement is still unclear. It is recommended to combine other brain imaging modalities methods with fNIRS at different measurement time points to further explore the underlying neural mechanisms of fNIRS-NFB for behavioral improvements, and fMRI might be the best choice for higher spatial resolution and subcortical imaging ability.

## Conclusion

In summary, the present pilot study demonstrates that real-time fNIRS-NFB training is feasible to allow participants to volitionally manipulate the neural activity in LPC. Successful modulation on LPC was accompanied by significantly increased spatial memory, and spatial memory changes can be predicted by NFB-induced activation changes in this region. Given the special advantages of fNIRS-NFB, this study has important implications for future possible applications in clinical settings or normal life situations for individuals with spatial memory problems. To achieve this goal, more research is needed in the future.

## Data Availability Statement

The raw data supporting the conclusions of this article will be made available by the authors, without undue reservation.

## Ethics Statement

The studies involving human participants were reviewed and approved by the Southwest University Brain Imaging Center Institutional Review Board. The patients/participants provided their written informed consent to participate in this study.

## Author Contributions

CZ and XH contributed to the conception and design of the study. XH, XX, and YG contributed to experimental design. XH collected experimental data, analyzed the experimental data, and wrote the draft of the manuscript. CZ and XH contributed to the interpretation and discussion of the results. All authors contributed to manuscript revision and approved the submitted version.

## Conflict of Interest

The authors declare that the research was conducted in the absence of any commercial or financial relationships that could be construed as a potential conflict of interest.

## Publisher’s Note

All claims expressed in this article are solely those of the authors and do not necessarily represent those of their affiliated organizations, or those of the publisher, the editors and the reviewers. Any product that may be evaluated in this article, or claim that may be made by its manufacturer, is not guaranteed or endorsed by the publisher.

## Footnotes

1
https://www.bcbl.eu/databases/multipic

2
www.psychtoolbox.org
